# Study of Geo-Electric Data Collected by the Joint EMSEV-Bishkek RS-RAS Cooperation: Possible Earthquake Precursors

**DOI:** 10.3390/e20080614

**Published:** 2018-08-18

**Authors:** Konstantina Papadopoulou, Efthimios Skordas, Jacques Zlotnicki, Toshiyasu Nagao, Anatoly Rybin

**Affiliations:** 1 Section of Solid State Physics, Department of Physics, National and Kapodistrian University of Athens, Panepistimiopolis, Zografos, Athens 157 84, Greece; 2 Solid Earth Physics Institute, Department of Physics, National and Kapodistrian University of Athens, Panepistimiopolis, Zografos, Athens 157 84, Greece; 3 CNRSDR7, Observatoire de Physique du Globe de Clermont-Ferrand, Laboratoire Magmas et Volcans, 5 rue Kessler, 63000 Clermont Ferrand, France; 4 Institute of Ocean Research and Development, Tokai University, Shizuoka 424-0902, Japan; 5 Research Station RAS, Bishkek-49, Kyrgyzstan 720049, Russia

**Keywords:** seismic electric signal, cross-correlation, anomalous telluric currents, resistivity, earthquakes

## Abstract

By employing the cross-correlogram method, in geo-electric data from the area of Kyrgyzstan for the period 30 June 2014–10 June 2015, we identified Anomalous Telluric Currents (ATC). From a total of 32 ATC after taking into consideration the electric current source properties, we found that three of them are possible Seismic Electric Signal (SES) activities. These three SES activities are likely to be linked with three local seismic events. Finally, by studying the corresponding recordings when a DC alternating source injects current into the Earth, we found that the subsurface resistivity seems to be reduced before one of these three earthquakes, but a similar analysis for the other two cannot be done due to their large epicentral distance and the lack of data.

## 1. Introduction

Seismic Electric Signals (SES) [1,2,3,4] are low frequency (≤1 Hz) transient changes of the electric field of the Earth that have been found to precede major earthquakes with lead times ranging from several hours to a few months [5,6]. They are emitted when the gradually increasing stress before an earthquake reaches a critical value [7], in which the electric dipoles formed due to point defects [8,9] in the future focal area exhibit cooperative orientation, thus resulting in an emission of a transient electric signal. SES can consist of a single pulse or a series of pulses. The latter are referred to as SES activities [6,7].

In Greece, practically, the potential difference ΔV is measured between two electrodes $Pb/PbCl_{2}$ placed 2 m [10] deep into the earth and which form a measuring dipole. There are two types of dipoles: the short dipoles with length *L* 50–400 m and the long dipoles with length 2–20 km [10]. At least four short dipoles should be installed perpendicular to each other in the directions east–west (EW) and north–south (NS) while there should not be any common electrodes. The places where the long dipoles are installed are selected to allow distinguishing the SES from nearby man-made noise sources [10,11,12,13].

Electric variations can be considered as SES if they satisfy the following four criteria simultaneously [6]:

- They appear selectively at some of the stations that constitute a recording network.
- They appear simultaneously at the long and short dipoles of the recording station.
- The ratio $\frac{\Delta V}{L}$ is constant for the short dipoles oriented in the same direction.
- The criterion $\frac{\Delta V}{L}\approx const$ must hold for a long and short dipole placed parallel to each other. That means that both the short and long dipoles record approximately the same mean value of the electric field.

The scope of this paper is to identify SES activities among Anomalous Telluric Currents (ATC) detected in geoelectrical data in Kyrgyzstan during our cooperation with EMSEV (Electromagnetic Studies of Earthquakes and Volcanoes) and RS-RAS (Bishkek Research Station of the Russian Academy of Science). ATC are either possible precursory electric currents flowing inside the earth or artificial noise originating from man-made sources. These can be distinguished by the aforementioned four criteria or by employing natural time analysis [10,14,15,16]. For SES activities, the entropy *S* in natural time χ defined as the derivative with respect to *q* of the fluctuation function $\langle \chi^{q}\rangle -{\langle \chi \rangle }^{q}$ at q=1, which results in: S≡〈χlnχ〉-〈χ〉ln〈χ〉, as well as the entropy $S_{-}$ which results when we employ time-reversal are both smaller than the entropy $S_{u}$ of the uniform distribution in natural time (see Ref. [17]). Notably, it has been recently shown in natural time that an SES activity initiates when the fluctuations of the order parameter of seismicity minimize [18] and in addition the change of the entropy of seismicity under time reversal exhibits a precursory minimum [19] with a lead time comparable to that of an SES activity. In other words, the entropy of seismicity in natural time exhibits a precursory behavior closely associated with the appearance of the SES.

According to the review article by Uyeda and coworkers [20], the earthquake prediction method based on SES, which is called VAN (VAN comes from the initials of Varotsos, Alexopoulos and Nomikos) method [21], has been a target of a heated debate (e.g., see Refs. [22,23,24]). As far as Uyeda and coworkers have critically examined, VAN successes are convincing (e.g., see Refs. [25,26,27]) and show in their Figure 4 are the score of VAN predictions for the period 1985–2003. They also state that public impact of VAN’s predictions has been large because lives have actually been saved at some disastrous earthquakes [28].

Preliminary results of this cooperation have been presented [29]. The present paper is a continuation of that study. Data from a longer period are examined. In particular, while only one case was briefly presented previously [29], here we examine the whole period 30 June 2014–10 June 2015 and identify a total of 32 electrical disturbances, of which three are possibly found to be SES activities. In the next section, a brief description of the methods used is provided. Our results are presented in Section 3 and a discussion follows in Section 4. Finally, in Section 5, we present our conclusions. Appendix A is also given where the Matlab code used here to produce cross-correlograms is provided.

## 2. Materials and Methods

### 2.1. Station Configuration and Data Recording

**Figure 1:**
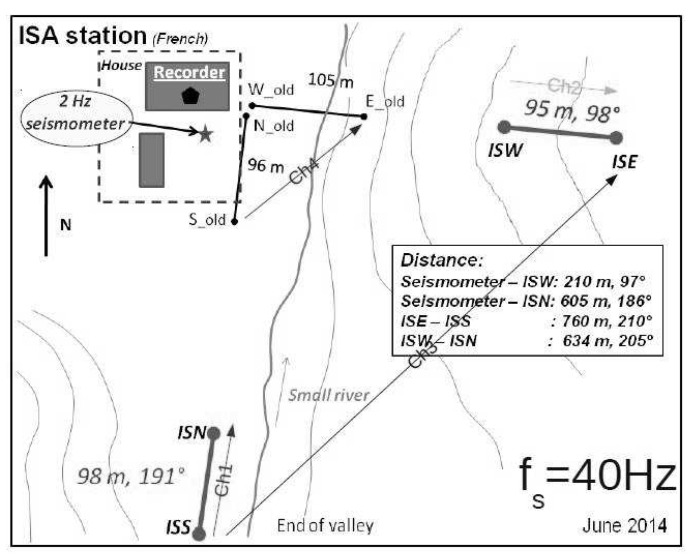
The configuration of the French autonomous station Issyk-Ata (ISA) as installed on 29 June 2014.

The autonomous French station designated as ISA (Issyk-Ata) is installed in the town of Bishkek at 42.64${}^{\circ }$ N, 74.96${}^{\circ }$ E. Its new configuration, updated on 29 June 2014, consists of two operating short dipoles (CH1, CH2) with lengths $L_{1}$ = 98 m and $L_{2}$ = 95 m, respectively, in the directions north–south (NS) and east–west (EW). A third, longer dipole (CH3) that passes under a small river records data with very small electric field amplitude due to a faulty electrode. The electrodes, 4 $Pb/PbCl_{2}$ are brought from France (0.0 mV between each of them), and buried at approximately 60 cm depth in wet soil with a little amount of salted water. The sampling frequency is $f_{s}$ = 40 Hz. Finally, a DC alternating source injects current into the Earth for 5 s, then inverses its polarity and continues to operate for another 5 s. Consequently, the period of the source is *T* = 10 s. At the end of each day, a data file is created containing the potential difference ΔV recordings of the day. The station’s configuration can be seen in Figure 1.

The data analyzed cover the period 30 June 2014–10 June 2015. We should note that the DC alternating source did not operate everyday during this period.

**Figure 2:**
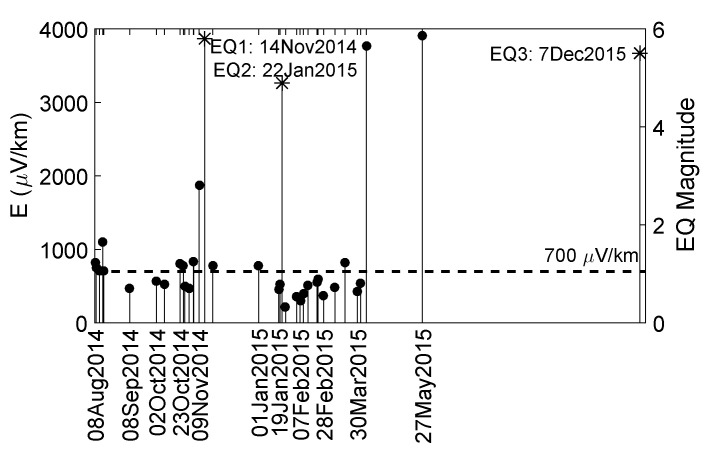
The ATC of Table 1 in chronological order and the earthquakes suspect for ATC, together with the total electric field recorded by the two channels of ISA (left y-axis, circled stems) and the earthquake magnitude (right y-axis, starred stems). A horizontal line has been drawn at 700 μV/km to separate the most prominent ATC.

The electric field *E* shown in Figure 2 is computed as follows:

CH1 records an electric field $E_{1}=\frac{\Delta V_{1}}{L_{1}}$ while CH2 records an electric field $E_{2}=\frac{\Delta V_{2}}{L_{2}}$. The two channels are perpendicular to each other so, assuming that *E* is homogeneous within the range of the recording site, the total electric field recorded is given as:

(1) $$E=\sqrt{{E_{1}}^{2}+{E_{2}}^{2}}$$

### 2.2. Cross-Correlogram Method Background

In Ref. [30], the term *correlogram* is used for displays showing periodicity characteristics of a voice. The authors’ aim was to compute the correlation (Pearson coefficient) between two time windows of the same signal to identify pathological traits in patients’ voices. Most recently, in Ref. [31], the correlogram method was also used to identify the periodicity of back pressure when a tube was submerged in water at various depths, during a voice therapy method where a patient phonated into the tube. The authors, while increasing the flow, identified from the correlograms three different patterns for the bubbles created into the water: regular (one by one), bimodal and chaotic. Their correlograms presented two candidates for the period of back pressure, thus, in general, the correlograms are able to display the periodicity of a signal with both regular and non-regular time period. Moreover, in Ref. [32], the correlogram was used to extract the fundamental frequency of the duplex strings of a piano which is not stable. The method allowed the authors to control their parameters (e.g., window length for correlation, number of periods from tone start, etc.) manually during the analysis. In general, the correlogram has been used in various fields such as neonatal pain in relation to delivery mode [33] and irregularities and disorders of the voice [34,35].

In the present paper, we propose the term *cross-correlogram* for the colored “maps” produced to display the cross-correlation between respective time windows of two signals. Details are given in Section 2.3. Our scope is to identify SES in geo-electric data which should appear at the same time at a station’s various dipoles (see Section 1), hence we need to examine the behavior of the channels’ recordings simultaneously.

### 2.3. The Cross-Correlogram Method for the Identification of ATC

If we have two time-series $x_{t}$ and $y_{t}$ of length *N* where $x_{t}$ is delayed by *T* samples (lag) with respect to $y_{t}$, we can compute their cross-covariance as:

(2) $$\sigma_{xy}\left(T\right)=\frac{1}{N}\sum_{t=1}^{N-T}\left(x_{t}-\mu_{x}\right)\left(y_{t+T}-\mu_{y}\right)$$

where $\mu_{x}$ and $\mu_{y}$ are the mean values of the time-series. The cross-covariance shows the tendency of the two time-series to change in a similar way. A normalized version of $\sigma_{xy}\left(T\right)$, which we use from now on, is the cross-correlation given in Equation (3):

(3) $$r_{xy}\left(T\right)=\frac{\sigma_{xy}\left(T\right)}{\sqrt{\sigma_{xx}\left(0\right)\sigma_{yy}\left(0\right)}}.$$

We compute the cross-correlation $r_{xy}\left(T\right)$ of Equation (3) between the two channels of ISA, CH1 and CH2, working as follows:

Firstly, we divide each channel’s data into non-overlapping windows with duration *t* = 300 s, i.e., $N=f_{s}\times t$ = 12,000 samples. This value is chosen because we expect to see enough pulses within five minutes so that a high correlation may be computed. Then, for each pair of windows, we compute $r_{xy}\left(T\right)$ using the Matlab function *crosscorr(X, Y, NumberOfLags)*. The variable *NumberOfLags* accepts any integer as input. However, to better represent the periodicity of the source in our figures, we choose NumberOfLags = 1200 samples here. *Crosscorr* will then compute 1200 lag values above the T=0 level and 1200 lag values below it. Taking under consideration the sampling frequency $f_{s}$ = 40 Hz, we essentially compute the cross-correlation for each pair of 300-s windows for lag values T∈[-30,30] s.

**Figure 3:**
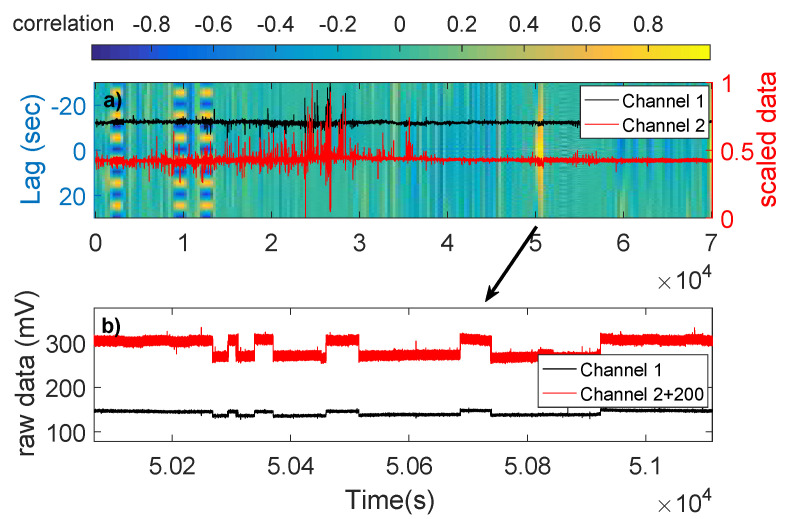
(**a**) The cross-correlogram of 27 May 2015 (the units in the horizontal axis are in $10^{4}$ s); and (**b**) the pulses found at the time of high correlation. The alternating yellow-blue areas in (**a**) show the periodicity of the source.

Finally, we draw a colored “map” of the $r_{xy}\left(T\right)$ values, hereafter referred to as cross-correlogram. Areas with high correlation (the pulses have the same polarity in the two channels) or high anti-correlation (the pulses have inverse polarity in the two channels) at T=0 are considered as suspect for ATC. The necessity of this color two-dimensional map arises from the presence of the following two origins: Besides a possible ATC, a periodic DC alternation source operates. This operating DC alternating source can be identified by the cross-correlogram in the same fashion as seeking for a “hidden frequency” in Ref. [30] (see the yellow and blue stripes in the leftmost side of Figure 3 that is discussed below). As shown in Ref. [36], the electric field of signals prior to major earthquakes has a slow and a fast component with a time difference of the order of some tenths of a second between them. Therefore, the reason we choose to examine the area of T=0 is the fact that, due to our high sampling frequency, there should not be any observable delay between the two channels’ recordings. We should note that not all high-correlated or high-anticorrelated areas depicted in the cross-correlograms contain ATC. Most of these areas contain either a level change or magnetotelluric disturbances. Therefore, we should examine all these correlated areas carefully in order to decide whether they contain an ATC [3,37,38,39,40,41].

### 2.4. The Earthquakes Possibly Preceded by SES

As a next step, we need to figure out which earthquakes that occurred in the general area of Kyrgyzstan could be candidates to be connected to a possible SES. The seismic catalogue we use is the one provided by USGS (United States Geological Survey, https://earthquake.usgs.gov/earthquakes/search/) in the period 30 June 2014–1 January 2016 (in accordance with our electric data and taking also under consideration the lead time of SES) and in the area 40–44${}^{\circ }$ N, 72–78${}^{\circ }$ E. In Table 2, we give all earthquakes with magnitude M4.9 or larger in this area. To judge which of these seven earthquakes tabulated may justify the appearance of SES, we rely on the rules emerged from the long term experimentation in Greece during 1980s and 1990s. These experimental rules are the following (see pp. 8–9 in Ref. [7]): for epicentral distances *r* of the order of a few tens of km, the SES are clearly seen for earthquake magnitude of at least 4.0. When *r* is around 100 km, the appropriate M value should be at least around 5.0–5.5. For even larger epicentral distances, i.e., around 150–200 km, the appropriate magnitude threshold should be close to 6.0. Thus, according to these rules, there exist three classes of earthquakes that may justify the appearance of SES. First, the nearby earthquakes, i.e., at distances a few tens of km from the recording station ISA: this is the case of the M4.9 earthquake on 22 January 2015. Second, the earthquakes with *r* around 100 km: this is the case of the M5.5 earthquake on 7 December 2015. Third, the earthquakes with *r* around 150–200 km and M close to 6.0: there exists only one earthquake, i.e., the one on 14 November 2014, with magnitude values M6.1 from Ref. [42] and M5.8 according to the local Kyrgyz Seismic Network (KNET). KNET instead of the magnitude *M* of the earthquake, reports its energy class *K* which provides an estimation of the radiated seismic energy from an earthquake and can be converted to magnitude using Equation (4) [43].

(4) $$M=\frac{K-4}{1.8}$$

The remaining two earthquakes in Table 2 have appreciably larger epicentral distances 340 km and 291 km and magnitudes markedly smaller than 6.0.

**Figure 4:**
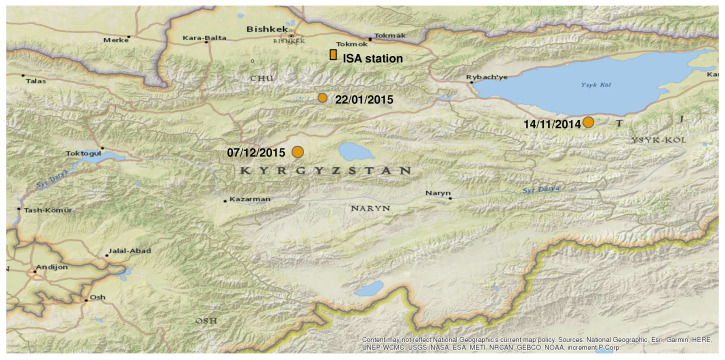
The epicentres of the earthquakes of Table 3 and the location of the ISA station (solid rectangle) on the ESRI Base Map as available in the ArcMap application.

Figure 4 shows the epicentres of the three earthquakes the magnitude M and the epicentral distance *r* of which may justify the appearance of SES according to the rules developed in Ref. [7] (see also Table 3).

## 3. Results

Table 1 shows the ATC identified after the cross-correlogram method (see Section 2.3) was applied to daily geo-electric recordings in the period 30 June 2014–10 June 2015.

Figure 2 shows the ATC of Table 1 in chronological order, together with the total electric field recorded by the two channels of ISA (see Section 2.1) and the three earthquakes suspect for SES (see Section 2.4).

### 3.1. Classification of ATC-Possible SES Activities

By observing Figure 2 and the corresponding cross-correlograms, we categorize those ATC of Table 1 which have the strongest electric field (above 700 μV/km, black horizontal line in Figure 2). This level of 700 μV/km is chosen as follows: we first consider the fact that the SES pulses’ amplitude is of the order of 1 mV/km. Secondly, we select the level so that the amplitude to exceed that of a reasonable number (i.e., at least a few) of the ATC identified. Note that, if we alternatively select the level to be 800 μV/km, our results here do not essentially change: instead of three SES, we would have only two because the SES on 9 August 2014 would be noise. The criterion we use for the classification of the ATC’s pulses as SES activities is that the ratio $\frac{{\frac{\Delta V_{2}}{L_{2}}}_{|EW}}{{\frac{\Delta V_{1}}{L_{1}}}_{|NS}}$ should be constant in all of the selected ATC’s pulses. The reason for that criterion is that, in the case of an SES, we do not expect the signal’s source to change its orientation. All the pulses identified are of boxcar shape. The candidate ATC belong to the following three types and are listed below:

- **Type 1**: 9 August 2014, 1 January 2015, and 27 May 2015. These ATC consist of rectangular pulses of almost the same amplitude.
- **Type 2**: 9 November 2014 and 21 November 2014. These ATC show some escalations, which means the recordings change levels constantly.
- **Type 3**: 23 October 2014 and 7 April 2015. These ATC show non-constant pulse amplitude.
- The ATC of 4 November 2014 is only partially recorded at one channel. If we examine a larger time-period than the 300-s windows of the cross-correlograms (see Section 2.3), we see that the ATC’s later pulses are only recorded at one of the two channels, therefore it cannot be an SES.
- **Single Pulses**: 8 August 2014, 12 August 2014, 15 August 2014, 16 August 2014, 26 October 2014, and 19 March 2015. Since they do not contain a number of pulses, we cannot consider them as SES activities.

**Figure 5:**
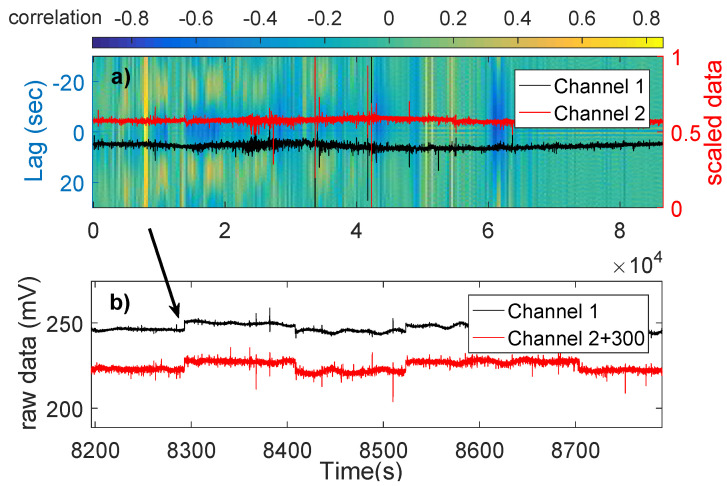
(**a**) The cross-correlogram of 9 August 2014 (The units in the horizontal axis are in $10^{4}$ s); and (**b**) the pulses found at the time of high correlation.

**Figure 6:**
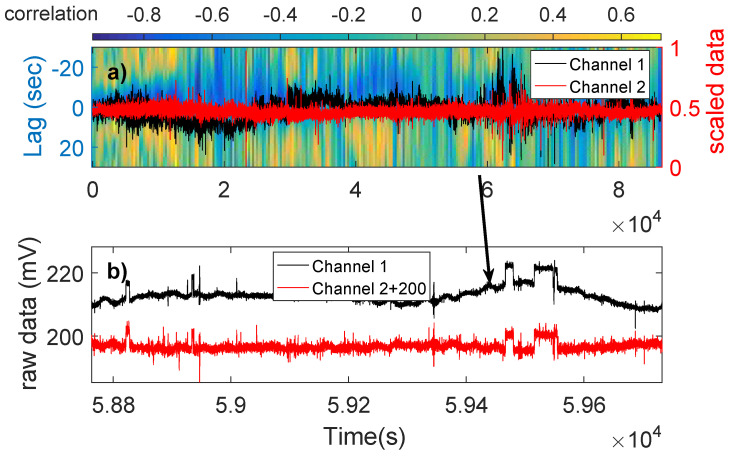
(**a**) The cross-correlogram of 1 January 2015 (the units in the horizontal axis are in $10^{4}$ s); and (**b**) the pulses found at the time of high correlation.

Only Type 1 can be considered as possible SES activities. Therefore, only three cases can be considered as SES activities, i.e., they have more than a single pulse: The one on 9 August 2014, almost three months before the 14 November 2014 earthquake (see Section 2.4) of magnitude M5.8, the one on 1 January 2015, 22 days before the M4.9 22 January 2015 earthquake (see Section 2.4), and the one on 27 May 2015, almost six months before the 7 December 2015 M5.5 earthquake (see Section 2.4). Figure 3, Figure 5 and Figure 6 show the cross-correlograms of these three cases.

We now confirm that the ratio $\frac{{\frac{\Delta V_{2}}{L_{2}}}_{|EW}}{{\frac{\Delta V_{1}}{L_{1}}}_{|NS}}$ is constant for each pulse of the three possible SES activities. The results are shown in Table 4, Table 5 and Table 6. By applying a paired t-test assuming ($H_{0}$ hypothesis) that the rise and decay values in each of the Table 4, Table 5 and Table 6 come from distributions with equal means, we found the following:

- **9 August 2014**: The *t*-test confirms the $H_{0}$ hypothesis with a *p*-value equal to 0.79.
- **1 January 2015**: The *t*-test confirms the $H_{0}$ hypothesis with a *p*-value equal to 0.92.
- **27 May 2015**: The *t*-test confirms the $H_{0}$ hypothesis with a *p*-value equal to 0.31.

Note: The *p*-value is the probability of obtaining a result by chance under the $H_{0}$ hypothesis. A small *p*-value (≤0.05) indicates that the $H_{0}$ hypothesis should be rejected.

Next, we examine the rise and decay times τ of the pulses of the three possible SES activities. We define the rise time as the time needed for the pulse to reach 85% of its maximum amplitude, starting to measure it from the time the pulse has reached 15% of its maximum amplitude [36]. In Table 7, we compile the mean values of the rise and decay times of the three possible SES activities, as well as the mean values of the rise and decay times of the source’s pulses recorded by the two channels of ISA station. The large values of the standard deviation σ in the 1 January 2015 case are due to an outlier in the rise time of the first pulse due to noise. If we exclude this value from the computations, we obtain much smaller mean and σ, as shown in Table 7. However, the same cannot be said for the third case of the 27 May 2015 ATC. Its large σ value as well as the application of a Kolmogorov–Smirnov test show that the rise time is not well defined. This fact leads us to suspect that the ATC of 27 May 2015 is due to a local source of noise and not to an actual SES activity where we expect an abrupt change of the electric field.

### 3.2. Subsurface Resistivity before Major Earthquakes

According to Ohm’s law, the electric current density *J* is related to the conductivity σ of the medium in which the current flows and the electric field *E* according to the equation J=σE, where $E=\frac{\Delta V}{L}$. For a change of the electric field and hence for ΔV, there should be either a change in the conductivity or in the current density.

**Figure 7:**
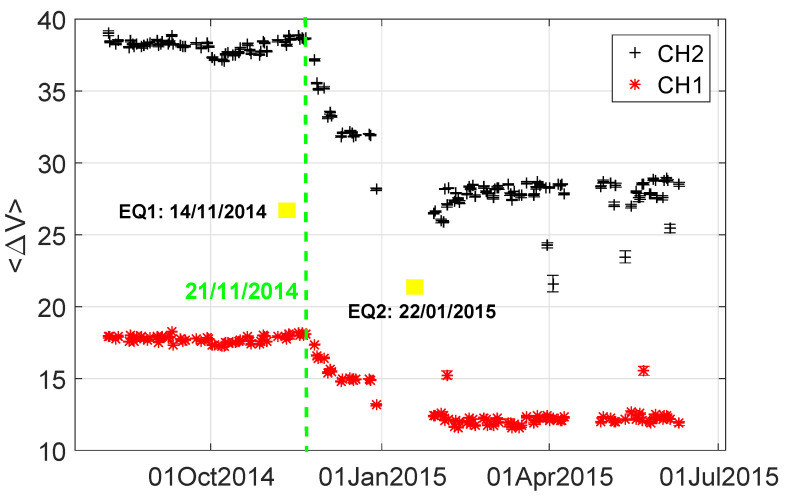
The daily mean of the potential difference recorded at the time the source operated at the two channels of ISA. The green vertical line is drawn at the time of the drop in 〈ΔV〉 (in mV) on 21 November 2014. The yellow rectangles are placed at the occurrence time of the two earthquakes.

**Figure 8:**
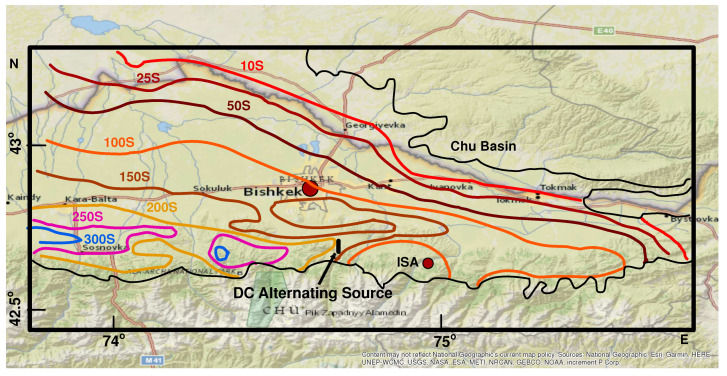
The profiles of subsurface conductivity in the Chu Basin in Kyrgyzstan. The arrow shows the position of the DC Alternating Source which injects 600 A at a distance of 4.3 km. Contours were taken from Figure 3 of Ref. [44] and are superimposed on the ESRI Base Map as available in the ArcMap application.

Figure 7 shows the daily mean of the potential difference 〈ΔV〉 computed by averaging the values of the potential difference of the source’s pulses during 24 h recorded at the time the source operates at the two channels of ISA (cf. Bishkek RS-RAS daily operates an electric DC alternating source with period 10 s of dipole moment 600 A × 4.3 km, the location of which is shown in Figure 8). There is a significant drop in ΔV on 21 November 2014, i.e., two months before the earthquake of 22 January 2015. Considering that the current density injected into the earth by the source is constant, this drop should be due to a rise in conductivity and, since the resistivity ρ is given as $\rho =\frac{1}{\sigma }$, the drop in 〈ΔV〉 corresponds to a drop in the subsurface resistivity which is stabilized after the earthquake. The same behavior cannot be observed for the earthquake on 14 November 2014, possibly due to its large distance from the recording station (see Table 3) while lack of data does not allow the same analysis for the earthquake on 7 December 2015.

**Figure 9:**
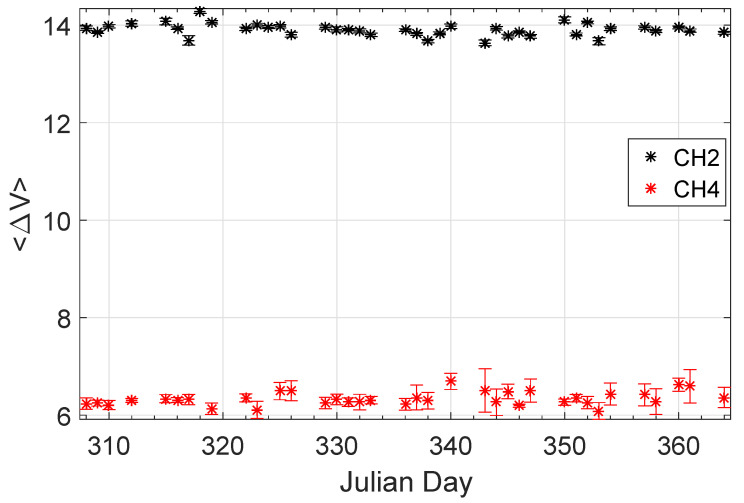
The daily mean 〈ΔV〉 (in mV)of the potential difference recorded at the time the source operated at the two channels of ISA for the year 2013 during the months November and December.

Finally, Figure 9 shows the daily mean of the potential difference recorded by two measuring dipoles (labeled channels CH2 and CH4 in Figure 9) at the time the source operated during November–December 2013 (at that time the station’s configuration was different from that depicted in Figure 1). An inspection of Figure 9 shows a stable behavior of both 〈ΔV〉 in 2013. Thus, the change in 〈ΔV〉 observed during November 2014 in Figure 7 does not appear during the same period in 2013, and hence the drop in subsurface resistivity before the earthquake of 22 January 2015 cannot be attributed to seasonal (weather) conditions.

## 4. Discussion

Our cross-correlograms are also able to show periodicity characteristics in the data. An example of that can be seen in Figure 3 where the periodicity of the source is displayed as alternating yellow-blue areas in the y-axis of Figure 3a. In the Appendix, we provide the Matlab code used to produce the cross-correlograms. The code is compatible with Matlab version R2016a.

With regards to the decrease in the subsurface resistivity two months before the M4.9 earthquake on 22 January 2015, similar results have been found in many studies. For example, Chu et al. [45], considering earthquakes within 200 km of their measuring sites, found that the resistivity began to drop two years before the M7.8 1976 Tangshan earthquake. The authors also claim that this change is not due to rainfall but it correlates with water level changes and follows the same pattern as the geologic and tectonic features of north China. Moreover, Kayal and Banerjee [46] recorded similar drops in resistivity before the M5.8 Cachar earthquake (31 December 1984) and before three earthquakes on July 1985. Although the authors also claim no correlation to rainfall, they showed that the orientation of the measuring dipoles plays a significant part in the detectability and behavior (increase or decrease) of the resistivity changes.

Concerning the rise time of the three possible SES activities reported in Table 7, it has been shown [36] that the rise time of an ATC pulse depends on the subsurface resistivity ρ according to the relation:

(5) $$\tau =\frac{\mu R^{2}}{4\rho }$$

where μ is the magnetic permeability and *R* is the distance from the source that emits the current. Here, we found that the rise times of the two possible SES activities of 9 August 2014 and 1 January 2015 (see Table 7) are smaller than the mean rise time of the source pulses. These results are compatible with the conductivity profiles of Figure 8 which show an increase in the resistivity (decrease of conductivity) south and southeast of ISA where the two corresponding earthquakes on 22 January 2015 and 14 November 2014 occurred (see Section 2.4.)

We clarify that the present method on cross-correlograms described in this paper is general, thus it can be applied to other areas as well.

## 5. Conclusions

Studying the almost one year (30 June 2014–10 June 2015) geo-electric data in the area of Kyrgyzstan, we found 32 ATC using the cross-correlogram method. Only three of them, observed on 9 August 2104, 1 January 2015 and 27 May 2015, have been identified as possible SES activities. The fact that only short dipoles have been used in this study did not allow us to apply the $\frac{\Delta V}{L}$ criterion, according to which an SES should have $\frac{\Delta V}{L}\approx const$ for a long and short dipole placed parallel to each other, to exclude the possibility of a surface source for these three ATC. However, the analysis of the rise time and the amplitude of the ATC pulses casts doubt in the classification of the ATC of 27 May 2015 as SES activity.

Finally, by studying source recordings, we found a decrease in the subsurface resistivity two months before a major earthquake in the area.

## Figures and Tables

**Table 1:** The ATC identified by applying the cross-correlogram method to all the daily data in the period 30 June 2014–10 June 2015, the potential differences $\Delta V_{1}$ and $\Delta V_{2}$ recorded at the two channels CH1 and CH2 of ISA (see Section 2), and the ratio of the electric fields recorded at these two channels. The estimation errors of columns two and three are computed using the standard error of the difference of means. If we denote as $\bar{x_{1}}$ the mean value of the maxima of the source’s pulses and as $\bar{x_{2}}$ the mean value of the minima of the source’s pulses, then the aforementioned error is computed as $SE_{\bar{x_{1}}-\bar{x_{2}}}=\sqrt{\frac{\sigma_{1}^{2}}{N_{1}}+\frac{\sigma_{2}^{2}}{N_{2}}}$ where $\sigma_{1}$, $\sigma_{2}$ are the corresponding standard deviations, $N_{1}$ the number of the maxima and $N_{2}$ the number of minima. The large differences in these estimation errors for different entries are due to large fluctuations in $N_{1}$ and $N_{2}$ since the source does not operate for the same duration every day.

Date	$\Delta V_{1}$ (mV)	$\Delta V_{2}$ (mV)	$\frac{{\frac{\Delta V_{2}}{L_{2}}}_{|\mathrm{EW}}}{{\frac{\Delta V_{1}}{L_{1}}}_{|\mathrm{NS}}}$
8 August 2014	5.23±0.16	5.95±0.25	1.18±0.06
9 August 2014	4.83±0.14	5.41±0.20	1.16±0.05
12 August 2014	5.08±0.15	4.68±0.22	0.95±0.05
15 August 2014	6.68±0.20	8.17±0.43	1.26±0.08
16 August 2014	5.16±0.19	4.52±0.35	0.90±0.08
8 September 2014	2.36±0.12	3.87±0.26	1.69±0.14
2 October 2014	-2.62±0.11	-4.77±0.29	1.88±0.14
9 October 2014	2.65±0.10	4.29±0.17	1.67±0.09
23 October 2014	3.86±0.23	6.66±0.30	1.78±0.13
26 October 2014	-4.34±0.26	-6.04±0.32	1.44±0.11
27 October 2014	-2.70±0.13	-3.97±0.31	1.52±0.14
31 October 2014	-2.57±0.133	-3.78±0.35	1.52±0.16
4 November 2014	1.32±0.10	-7.81±0.23	-6.09±0.49
9 November 2014	-4.89±0.20	-17.11±0.47	3.61±0.18
21 November 2014	1.93±0.07	7.17±0.18	3.83±0.17
1 January 2015	5.62±0.16	5.05±0.23	0.93±0.05
19 January 2015	3.25±0.11	2.94±0.09	0.93±0.04
20 January 2015	3.71±0.14	3.39±0.16	0.94±0.06
25 January 2015	1.52±0.04	1.46±0.06	0.99±0.05
4 February 2015	-3.17±0.09	-1.29±0.02	0.42±0.01
7 February 2015	2.15±0.08	1.88±0.09	0.90±0.05
10 February 2015	-3.04±0.12	-2.35±0.14	0.80±0.06
14 February 2015	3.48±0.05	3.48±0.07	1.03±0.03
22 February 2015	-3.69±0.10	-3.89±0.13	1.09±0.05
23 February 2015	-4.60±0.05	-3.40±0.08	0.76±0.02
28 February 2015	-2.87±0.03	-2.07±0.04	0.74±0.02
10 March 2015	-3.25±0.03	-3.41±0.04	1.08±0.02
19 March 2015	-5.37±0.04	-5.80±0.07	1.11±0.02
30 March 2015	3.00±0.03	2.76±0.05	0.95±0.02
2 April 2015	4.21±0.02	3.07±0.04	0.75±0.01
7 April 2015	-8.64±0.08	-34.75±0.17	4.15±0.04
27 May 2015	-9.53±0.11	-35.96±0.43	3.89±0.07

**Table 2:** All earthquakes with magnitude 4.9 or larger from USGS within (40–44)${}^{\circ }$ N, (72–78)${}^{\circ }$ E.

Date	Time	Lat. (${}^{\circ }$)	Long. (${}^{\circ }$)	K	M	r (km)
15 August 2014	21:42:30.75	42.94	77.43	-	5 (mb)	204.47
14 November 2014	1:24:16.48	42.14	77.23	14.43	5.2 (mww)	194.24
					5.8 (KNET)	
					6.1 (Mpv) [42]	
10 January 2015	06:51:02.61	40.11	77.26	-	5.1 (mb)	340.33
22 January 2015	15:52:30.71	42.36	74.95	-	4.9 (mb)	30.92
17 November 2015	17:29:26.29	40.38	73.20	-	5.6 (mww)	290.97
1 December 2015	06:13:43.36	41.38	73.26	-	5.1 (mb)	198.82
7 December 2015	08:30:56.73	41.73	74.61	-	5.5 (mb)	105.22

**Table 3:** The earthquakes during the time period 30 June 2014–1 January 2016 and in the area 40–44${}^{\circ }$ N, 72–78${}^{\circ }$ E the magnitude M and the epicentral distance *r* of which may justify the appearance of SES according to the rules developed in Ref. [7].

Date	Time	Lat. (${}^{\circ }$)	Long. (${}^{\circ }$)	K	M	r (km)
14 November 2014	1:24:16.48	42.14	77.23	14.43	5.8 (KNET)	194.24
22 January 2015	15:52:30.71	42.36	74.95	-	4.9 (mb)	30.92
7 December 2015	08:30:56.73	41.73	74.61	-	5.5 (mb)	105.22

**Table 4:** The ratio $\frac{{\frac{\Delta V_{2}}{L_{2}}}_{|EW}}{{\frac{\Delta V_{1}}{L_{1}}}_{|NS}}$ for each pulse of the ATC of 9 August 2014.

No. of Pulse	Type	$\frac{{\frac{\Delta V_{2}}{L_{2}}}_{|\mathrm{EW}}}{{\frac{\Delta V_{1}}{L_{1}}}_{|\mathrm{NS}}}$
1	rise	1.16±0.05
1	decay	1.38±0.08
2	rise	1.15±0.06
2	decay	1.06±0.06
3	rise	1.09±0.10
3	decay	1.07±0.08
4	rise	1.12±0.11
4	decay	1.09±0.08

**Table 5:** The ratio $\frac{{\frac{\Delta V_{2}}{L_{2}}}_{|EW}}{{\frac{\Delta V_{1}}{L_{1}}}_{|NS}}$ for each pulse of the ATC of 1 January 2015.

No. of Pulse	Type	$\frac{{\frac{\Delta V_{2}}{L_{2}}}_{|\mathrm{EW}}}{{\frac{\Delta V_{1}}{L_{1}}}_{|\mathrm{NS}}}$
1	rise	0.93±0.05
1	decay	0.87±0.03
2	rise	0.86±0.04
2	decay	0.98±0.06
3	rise	0.93±0.06
3	decay	0.96±0.07
4	rise	0.93±0.05
4	decay	0.96±0.04
5	rise	1.08±0.05
5	decay	0.98±0.04
6	rise	0.94±0.11
6	decay	0.94±0.04

**Table 6:** The ratio $\frac{{\frac{\Delta V_{2}}{L_{2}}}_{|EW}}{{\frac{\Delta V_{1}}{L_{1}}}_{|NS}}$ for each pulse of the ATC of 27 May 2015.

No. of Pulse	Type	$\frac{{\frac{\Delta V_{2}}{L_{2}}}_{|\mathrm{EW}}}{{\frac{\Delta V_{1}}{L_{1}}}_{|\mathrm{NS}}}$
1	decay	3.89±0.07
1	rise	3.50±0.23
2	decay	3.40±0.32
2	rise	3.44±0.28
3	decay	3.98±0.35
3	rise	3.68±0.19
4	decay	3.84±0.13
4	rise	4.16±0.09
5	decay	3.95±0.09
5	rise	3.92±0.08
6	decay	3.87±0.11
6	rise	3.87±0.09
7	decay	4.59±0.35
7	rise	3.84±0.28
8	decay	3.72±0.25
8	rise	3.80±0.09

**Table 7:** The mean value of the rise and decay times for the pulses of the three possible SES activities, as well as those of the source.

Case	$<\tau_{\mathrm{CH}1}>$±σ (ms)	$<\tau_{\mathrm{CH}2}>$±σ (ms)
9 August 2014	48±14	39±14
1 January 2015	58±46	58±50
1 January 2015 (excl. outlier)	45±12	44±17
27 May 2015	61±58	79±47
source	68±5	70±2

## Abbreviations

The following abbreviations are used in this manuscript:

ATC	Anomalous Telluric Currents
SES	Seismic Electric Signals
EW	East–West
NS	North–South
EMSEV	Electromagnetic Studies of Earthquakes and Volcanoes
RS-RAS	Bishkek Research Station of the Russian Academy of Science
ISA	Issyk Ata
USGS	United States Geological Survey
KNET	Kyrgyz Seismic Network

## Appendix A. Matlab Code for Cross-Correlograms

clear all; clc;

data=load(’ISN14296.dat’);

%the channels

x=data(:,2);

y=data(:,3);

%z=data(:,4);

%w=data(:,5);

fs=40; %the sampling rate for the raw data

% time data

datatime=[1:length(x)]; %number of samples which I turn into seconds below (x,y,z or w)

time=datatime/fs;

%window the data with t=300secs

t=300;  %in seconds

N=fs*t;  %the number of samples for t

%y = buffer(x,n) partitions a length-L signal vector x into nonoverlapping

%data segments (frames) of length n.

%Each data frame occupies one column of matrix output y

X=buffer(x,N);

Y=buffer(y,N);

%%%%%%%%%%%%%%%%%%%%%%%%%%%%%%%%%%%%%%%%%%%%%%%%%%%%%%%%%%%%%%%%%%%%%%%%%%%

% I want the cross-correlation between the columns of X-Y (the 1st column

% of X with the 1st column of Y etc)

%cross correlation column-wise:

j=1;

s=size(X);

col=s(2);

for i=1:col

  r(j,:)=crosscorr(X(:,i),Y(:,i),1200);

  j=j+1;

end

r=r’;

p=size(r);

maxlag=p(1)/2;

yaxisscale=[(-maxlag/fs):(maxlag/fs)]; %in seconds

%%%%%%%%%%%%%%%%%%%%%%%%%%%%%%%%%%%

figure

%plots

subplot (2,1,1)

yyaxis left

imagesc(time, yaxisscale, r)

%axis ([0,max(time),-maxlag/fs,maxlag/fs]);

ylabel(’Lag (sec)’)

handl = colorbar(’NorthOutside’);

ylabel(handl, ’correlation’)

hold on

yyaxis right

right_color = [1 0 0];  %red

ax = gca;

ax.YColor = right_color;

%scale it in the interval [0,1]

x=(x-min(x))/(max(x)-min(x));

plot(time,x,’k’)

hold on

y=(y-min(y))/(max(y)-min(y));

plot(time,y,’r’)

%xlabel(’Time(sec)’)

ylabel(’scaled data’)

legend (’Channel 1’, ’Channel 2’)

axis tight

subplot (2,1,2)

plot(time,data(:,2),’k’)

hold on

plot(time,data(:,3)+250,’r’)

xlabel(’Time(sec)’)

ylabel(’raw data (mV)’)

legend (’Channel 1’, ’Channel 2+250’)

axis tight
